# Performance, Recalibration, and Implementation of In-Hospital Mortality Prediction Models for Myocardial Infarction

**DOI:** 10.31083/RCM51293

**Published:** 2026-10-21

**Authors:** Erkun Xing, Yurun Su, Zekun Lang, Yongling Wa, Wei Hu, Xiaojuan Wang, Youqi Zhu, Jing Zhao, Ming Bai

**Affiliations:** ^1^The First School of Clinical Medicine, Lanzhou University, 730000 Lanzhou, Gansu, China; ^2^Heart Center, The First Hospital of Lanzhou University, 730000 Lanzhou, Gansu, China; ^3^Gansu Province Clinical Research Center for Cardiovascular Diseases, 730000 Lanzhou, Gansu, China

**Keywords:** acute myocardial infarction, hospital mortality, machine learning

## Abstract

Acute myocardial infarction remains a leading cause of cardiovascular mortality worldwide. Although reperfusion therapy has improved short-term outcomes, in-hospital mortality remains high, particularly among patients with complex conditions. Therefore, accurate mortality prediction models are essential for optimizing clinical decision-making and individualized management. This review summarizes the evolution of these models over the past two decades, from traditional statistical methods to machine learning. Indeed, machine-learning models may achieve slightly higher discrimination than traditional statistical models in selected datasets, particularly when high-dimensional and nonlinear clinical information is available. However, the magnitude of improvement is often incremental rather than transformative, and higher area under the curve (AUC) values do not necessarily indicate better calibration, interpretability, transportability, or clinical utility. Multicenter external validation studies indicate that performance is influenced by population heterogeneity, evolving treatment strategies, and data quality, with notable variation across subgroups, including Asian patients, women, and patients with diabetes. Recalibration is key to improving transportability and can be achieved through intercept adjustment, slope calibration, or model reconstruction; dynamic updating may help maintain long-term performance. Inadequate data standardization, concerns regarding black-box models, workflow misalignment, and limited policy support can all constrain clinical implementation. Therefore, this study proposes a technology–clinical–management pathway: promoting standardized data and federated learning, building lightweight interpretable embedded decision support, establishing model registration/certification and reimbursement linkages, and strengthening multidisciplinary training. Future work should prioritize multimodal data fusion, real-time prediction, improved interpretability, multicenter prospective validation, and cost-effectiveness assessment to support safe implementation across diverse healthcare systems. Notably, China represents an important regional context for evaluating these strategies.

## 1. Introduction

Acute myocardial infarction (AMI) is an acute cardiovascular event characterized by a sharp reduction or interruption of coronary blood supply, leading to ischemic and hypoxic necrosis of cardiac cells. The Global Burden of Disease study indicates that the occurrence rate of AMI is increasing annually, particularly against the backdrop of an aging society and lifestyle changes, thereby imposing growing pressure on public health systems [1]. In-hospital mortality is a key indicator for assessing the short-term prognosis of AMI patients, with reported rates varying across studies. For instance, data from the China Acute Myocardial Infarction Registry (CAMI) show that the in-hospital mortality rate for AMI patients is approximately 4.4%, while for patients complicated with cardiogenic shock (CS), this rate can be as high as 27.33% [2,3,4,5]. Furthermore, during the COVID-19 pandemic, the in-hospital mortality rate for AMI patients with concomitant COVID-19 infection was significantly elevated, increasing more than fivefold compared to non-infected patients, further highlighting the complexity of AMI clinical management [6]. The clinical management of AMI faces multiple challenges. Firstly, patient clinical manifestations are highly heterogeneous; some patients may lack typical chest pain symptoms, leading to diagnostic delays [7]. Secondly, the timing and quality of reperfusion therapy directly impact patient prognosis, yet in clinical practice, treatment delays frequently occur due to factors such as transfer delays and uneven distribution of medical resources [8]. Additionally, AMI patients often present with multiple underlying conditions, such as diabetes and chronic renal failure. These comorbidities not only complicate treatment but also significantly elevate the risk of in-hospital mortality. For example, the risk of in-hospital death for diabetic patients following AMI is 1.45 times higher than that for non-diabetic patients, while the risk for patients with chronic renal failure increases by 2.09 times [9,10]. Concurrently, the early identification and intervention of AMI complications, such as CS and multiple organ dysfunction syndrome, remain challenging in clinical management. Complications such as cardiogenic shock and multiple organ dysfunction are strongly associated with poor in-hospital outcomes in patients with AMI [11].

To address these challenges, there is an urgent need in clinical practice for efficient and precise risk assessment tools to achieve early patient stratification and individualized management. In-hospital mortality prediction models can assist clinicians in identifying high-risk individuals early after admission, thereby enabling the prioritization of medical resources and the implementation of more aggressive treatment strategies. For instance, for high-risk patients, intensive antiplatelet therapy, mechanical circulatory support, or emergency percutaneous coronary intervention (PCI) can be initiated early to reduce mortality risk. Studies have shown that simple scoring systems based on indicators such as age, heart rate, and systolic blood pressure, like the Thrombolysis In Myocardial Infarction (TIMI) score and Global Registry of Acute Coronary Events (GRACE) score, are widely used in clinical practice, but their predictive performance varies across different populations [12,13,14,15]. In recent years, machine-learning models have been increasingly used for outcome prediction after AMI because they can incorporate high-dimensional clinical variables and capture nonlinear relationships; however, available comparisons suggest that their improvement over conventional statistical models is often modest, context-dependent, and limited by insufficient external validation. For example, in AMI patients undergoing primary PCI, Achmad et al. [16] reported that a random forest (RF) model achieved an area under the curve (AUC) of 0.976 for predicting in-hospital mortality, compared with 0.973 for logistic regression. Although both models showed excellent discrimination, the absolute difference was minimal, suggesting that machine-learning approaches do not always provide clinically meaningful improvement over well-specified conventional models. Therefore, model evaluation should not rely solely on discrimination metrics, but should also consider calibration, interpretability, external validation, and feasibility of clinical implementation [16,17,18]. Recent biomarker-based studies also support this point; for example, the hemoglobin–albumin–lymphocyte–platelet score and admission serum malondialdehyde levels have been associated with in-hospital mortality in ST-segment elevation myocardial infarction (STEMI) or acute coronary syndrome (ACS) patients, suggesting that simple and interpretable variables may complement complex prediction models [19,20].

Despite significant progress in the development of in-hospital mortality prediction models for AMI, existing studies still face numerous methodological and practical challenges [21,22,23]. The methodologies for constructing these models vary widely, encompassing traditional statistical approaches such as logistic regression and Cox proportional hazards models, as well as machine learning techniques including RF, Support Vector Machines, and deep learning [24,25,26,27,28]. The predictive performance of these different methods varies; machine-learning models often demonstrate higher predictive accuracy but may be prone to overfitting and generally exhibit poorer interpretability [18,28,29]. Furthermore, there is considerable heterogeneity in the predictor variables incorporated into these models. Some models rely solely on clinical features, while others integrate laboratory parameters, imaging data, and genetic biomarkers, which compromises the comparability and generalizability of the models (Fig. 1) [25].

**Fig. 1.**
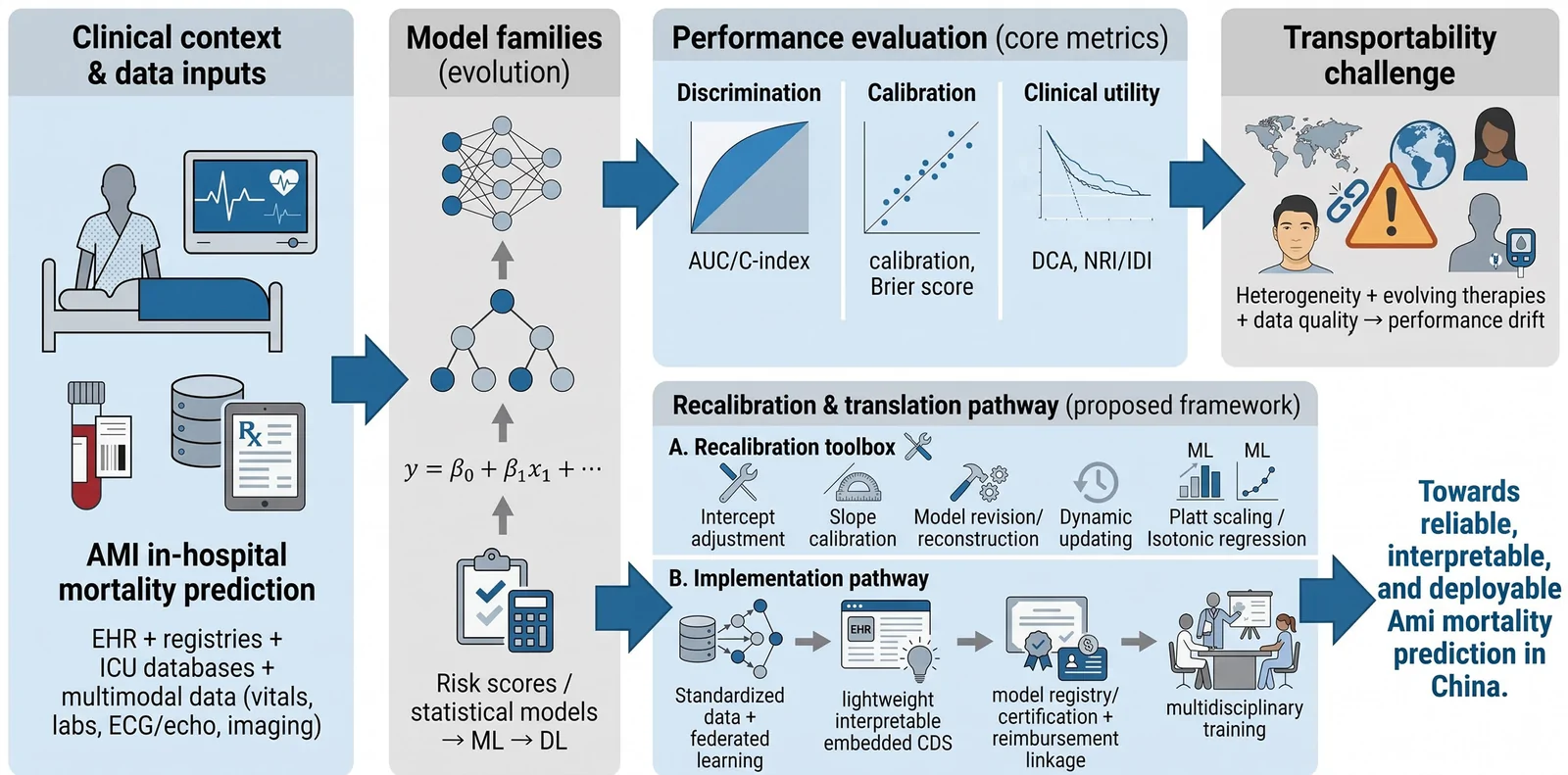
**Conceptual roadmap for in-hospital mortality prediction modeling in AMI**. EHR, electronic health records; ICU, intensive care unit; ECG, electrocardiography; ML, machine learning; DL, deep learning; AUC, area under the curve; DCA, decision curve analysis; NRI, net reclassification index; IDI, integrated discrimination improvement index; CDS, clinical decision support; AMI, acute myocardial infarction.

This manuscript is a narrative review. Therefore, a formal PRISMA flow diagram, quantitative synthesis, and risk-of-bias assessment were not performed. To enhance transparency, we searched PubMed, Embase, and Web of Science for English-language studies published between January 2000 and March 2026. The search terms included “acute myocardial infarction”, “in-hospital mortality”, “mortality prediction”, “risk score”, “machine learning”, “deep learning”, “external validation”, “calibration”, “recalibration”, and “clinical application”. Relevant references from key original studies and reviews were also manually screened.

We prioritized studies that reported the development, validation, comparison, recalibration, or clinical application of models for predicting in-hospital or short-term mortality in AMI. Articles were excluded if they were unrelated to AMI, lacked model performance information, or focused only on long-term prognosis without relevance to in-hospital prediction. The final selection was guided by relevance to the review topic rather than exhaustive systematic screening.

This review adopts a global perspective. It summarizes prediction models developed and validated across different countries and healthcare systems. China is discussed as a representative regional implementation context because of its large AMI burden, heterogeneous medical resources, and increasing availability of registry-based and electronic health record data.

## 2. Development of In-Hospital Mortality Prediction Models for AMI

As an acute and critical condition among cardiovascular diseases, AMI has long been associated with a high in-hospital mortality rate, posing a serious threat to patient life and health. To address this clinical challenge, the development of in-hospital mortality prediction models has evolved from a dominance of traditional statistical methods to the gradual application of machine learning and deep learning techniques. The core objective remains to provide a scientific basis for clinical decision-making through accurate risk stratification. The development of early models can be traced back to risk assessment tools based on expert experience and univariate analysis, such as the classic TIMI risk score and GRACE score. By integrating clinical variables including age, heart rate, blood pressure, and cardiac enzyme levels, these models employed traditional statistical methods like logistic regression to construct predictive equations, laying the foundation for risk stratification in AMI patients. With the accumulation of clinical data and advances in statistical methods, models gradually progressed toward multifactorial and multicenter approaches. For instance, a risk model developed based on the ACTION Registry-GWTG database incorporated data from over 240,000 patients. Through hierarchical logistic regression analysis, it identified independent predictors such as age, heart rate, systolic blood pressure, and history of cardiac arrest, achieving a concordance statistic (C-statistic) of 0.88, demonstrating good discriminative ability [30]. Concurrently, models targeting specific populations began to emerge. For example, the Women Acute Myocardial Infarction in-Hospital Mortality score (WAMI) for female AMI patients incorporated eight variables including age, systolic blood pressure, heart rate, and estimated glomerular filtration rate. In external validation, it achieved a C-statistic of 0.87, significantly outperforming the GRACE and TIMI scores [31].

Since the beginning of the 21st century, with the widespread adoption of electronic health records (EHR) and advancements in big data technology, ML methods have been increasingly applied to predict in-hospital mortality in AMI. These models may capture complex predictor interactions, but their incremental clinical value remains dependent on validation quality and implementation context. For instance, a model based on the RF algorithm achieved an AUC of 0.976 in predicting in-hospital mortality among AMI patients after PCI, but its performance was very close to that of the logistic regression model, indicating that the incremental gain in discrimination may be limited [16]. Similarly, the CatBoost model showed favorable discrimination, with an AUC of 0.92 for predicting in-hospital mortality in AMI patients with comorbid diabetes mellitus; however, its broader applicability requires further validation across independent populations and clinical settings [32]. Furthermore, deep learning techniques have shown potential in selected datasets, particularly when large-scale or multimodal data are available, although their incremental benefit over simpler models remains dependent on data quality, validation design, and clinical context. For example, a study based on the MIMIC-IV database showed that a deep neural network (DNN) achieved an AUC of 0.95 in predicting in-hospital mortality among elderly ST-segment elevation myocardial infarction (STEMI) patients, significantly surpassing the traditional TIMI risk score [33]. As shown in Table 1 (Ref. [33,34,35,36,37,38,39,40,41,42,43]), the in-hospital mortality risk prediction models for AMI can be broadly categorized into two main types: traditional statistical models and machine-learning models. Overall, machine-learning and deep learning approaches may offer methodological flexibility, but their clinical value depends on data quality, calibration, external validation, interpretability, and implementation feasibility. In contrast, traditional regression models are easier to interpret and apply at the bedside, although they may be less capable of modeling complex interactions. During clinical implementation, it remains essential to emphasize model recalibration and the evaluation of cross-center generalization capability.

**Table 1.** **Mainstream models for predicting in-hospital mortality in AMI: classes and key characteristics**.

Model class	Typical algorithms	Key characteristics	Typical data	Example performance	Main caveats
Traditional statistics	Logistic regression; Cox	Clinically guided predictor selection; linear predictor; outputs OR/HR	Structured clinical/lab variables; time-to-event endpoints for Cox	HAMIOT score: C-statistic 0.88 (train)/0.82 (valid) [34] CAMI score: C-statistic 0.84 (valid) [35]	Assumptions (e.g., linearity; proportional hazards); limited nonlinearities/interactions; often needs recalibration
	Nomogram (regression-based)	Visualizes coefficients as point scales; supports bedside individualized risk	Structured variables; simplified bedside scoring	AMI + HF nomogram: AUC 0.882 [36] SWEDEHEART nomogram: C-statistic 0.80 [37]	Still regression-based; performance depends on external validation and calibration
Machine learning	Random forest (RF)	Bagging ensemble of trees; captures nonlinearities/interactions; SHAP can aid attribution	High-dimensional structured predictors; mixed variable types	MIMIC-IV (AMI + diabetes): AUC 0.823 [38] STEMI 30-day death: AUC 0.804 [39]	Interpretability lower than regression; sensitive to missingness/data quality; requires tuning
	Gradient boosting trees (XGBoost; LightGBM)	Sequential boosting with optimized loss and regularization; often high accuracy	High-dimensional structured data	LightGBM (post-PCI AMI): AUC 0.93 [40] XGBoost (AMI + cardiogenic shock): AUC 0.869 [41]	Hyperparameter sensitivity; risk of information leakage; external validation required
	Neural networks (DNN/CNN)	Deep nonlinear mapping; supports multi-modal inputs (e.g., echo/imaging + labs)	Large datasets; imaging/echocardiography; multi-modal data	Echo DNN: AUC 0.912 [42] MIMIC-IV (elderly STEMI): AUC 0.95 [33]	High data/compute demand; weaker interpretability; overfitting risk
	NLP + deep learning	Extracts features from unstructured EHR text; can be fused with structured features	Free-text notes/reports; hybrid structured + text	Text mining to enrich predictors from EHR [43]	Generalizability across hospitals and languages; annotation/noise; privacy and governance

AMI, acute myocardial infarction; PCI, percutaneous coronary intervention; OR, odds ratio; HR, hazard ratio; AUC, area under the receiver operating characteristic curve; C-statistic, concordance statistic; RF, random forest; DNN, deep neural network; CNN, convolutional neural network; SHAP, Shapley additive explanations; EHR, electronic health record; NLP, natural language processing; STEMI, ST-segment elevation myocardial infarction; HF, heart failure.

In recent years, the development of predictive models has exhibited the following trends. First, there has been a shift from predicting a single outcome to predicting multiple outcomes. For instance, the KAMIR-NIH model simultaneously predicts the risks of in-hospital mortality and major bleeding, providing more comprehensive information for clinical decision-making [44]. Second, there is a transition from “black-box” models towards interpretable models. Methods such as Shapley additive explanations (SHAP) and LIME can partially improve the interpretability of ML models, but they do not fully eliminate the black-box nature of complex algorithms and should be regarded as supportive rather than definitive explanations [38]. Third, models are evolving from static to dynamic. For example, real-time prediction models based on continuously monitored data can promptly update a patient’s risk assessment [45] (Fig. 2).

**Fig. 2.**
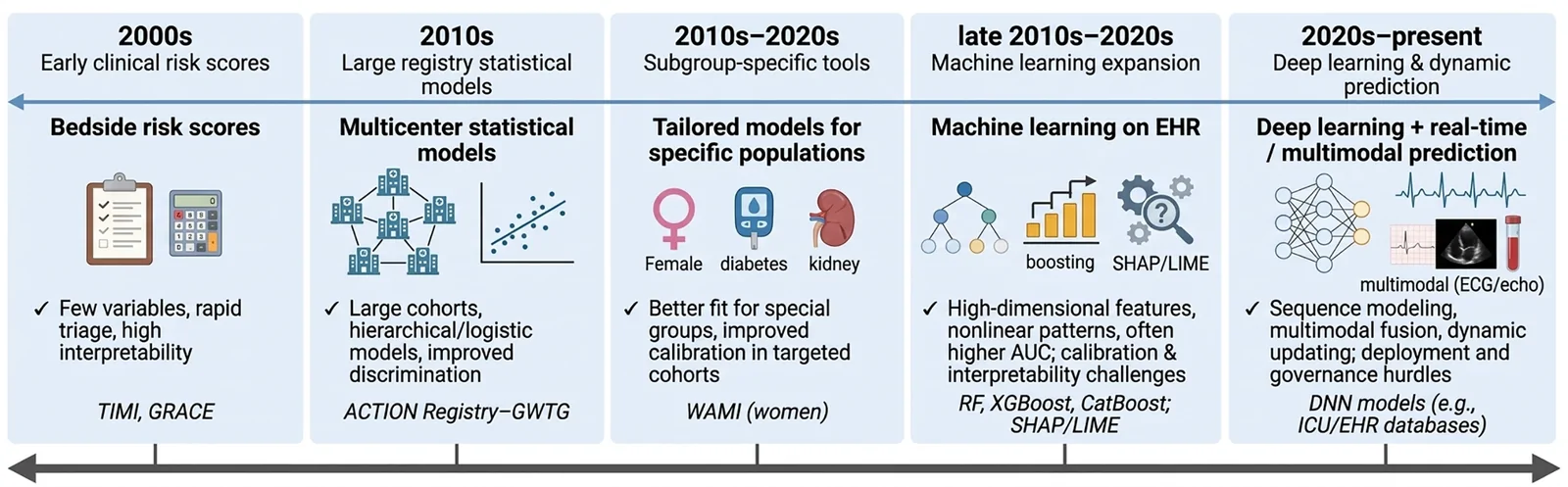
**Evolution of models for predicting in-hospital mortality in AMI**. TIMI, Thrombolysis In Myocardial Infarction; GRACE, Global Registry of Acute Coronary Events; ACTION, Acute Coronary Treatment and Intervention Outcomes Network; GWTG, Get With The Guidelines; WAMI, Women Acute Myocardial Infarction in-Hospital Mortality score; LIME, Local Interpretable Model-agnostic Explanations.

## 3. Comparison of In-Hospital Mortality Prediction Models

### 3.1 Cross-Sectional Comparison of Discrimination and Calibration

Traditional statistical models, such as logistic regression and the Cox proportional hazards model, are widely used in clinical practice due to their simplicity and interpretability. Their performance varies depending on model design, included variables, and the validation population. The GRACE score, one of the most globally utilized risk scores for acute coronary syndrome (ACS), has demonstrated good discriminative ability in multiple studies. In the Thai PCI registry study, the GRACE score achieved an AUC of 0.8957 for predicting in-hospital mortality, with a Hosmer-Lemeshow (H-L) test *p*-value of 0.036, indicating acceptable calibration [46]. In a Chinese ACS patient cohort, the GRACE score had an AUC of 0.926, which was slightly lower than that of the ACTION model, but exhibited superior calibration (H-L test *p*-value closer to 0.05) [47]. However, the performance of the GRACE score may be limited in specific subgroups. For instance, a study on patients with end-stage renal disease (ESRD) and AMI reported an AUC of only 0.754 for the GRACE score, which was significantly lower than that of a novel risk score developed based on variables such as C-reactive protein (CRP), left ventricular ejection fraction (LVEF), and age (AUC = 0.895) [48]. The ACTION Registry-GWTG risk score has shown excellent discriminative ability in large-scale cohorts. In the ACTION Registry-GWTG study, the model achieved a C-statistic of approximately 0.88 for predicting in-hospital mortality in patients with AMI [30]. In the aforementioned Chinese ACS cohort, the ACTION model achieved a high AUC of 0.945. Although its calibration was slightly inferior to that of the GRACE score (H-L test *p*-value < 0.05), its discriminative advantage was significant [47]. Traditional models developed for specific subgroups often exhibit more precise performance. For example, the WAMI score for female AMI patients demonstrated an external validation AUC of 0.87, which was significantly higher than that of the GRACE score (0.77) and the TIMI score (0.72) [31]. A prediction model for AMI patients complicated by CS achieved an AUC of 0.941 in the development set and 0.981 in the validation set, with its calibration curve nearly overlapping the ideal diagonal line.

Machine-learning models (e.g., RF, XGBoost, Bagging, neural networks), by processing high-dimensional data and complex nonlinear relationships, often outperform traditional models in terms of discrimination; however, their calibration and interpretability still require optimization. The RF model is one of the most frequently applied machine-learning algorithms for mortality prediction in AMI; however, reported performance varies substantially across datasets and should be interpreted in light of sample size, predictor availability, validation strategy, and risk of overfitting. For instance, an RF model developed for AMI patients with diabetes mellitus (DM) achieved an AUC of 0.823 on the validation set, which was significantly higher than that of the K-Nearest Neighbors (KNN) (0.751) and Decision Tree (0.747) models [38,49]. In a study on stacking models for non-ST-segment elevation myocardial infarction (NSTEMI) patients, a two-layer stacking model using RF as the base learner achieved a high AUC; however, the complexity of ensemble models increases the need for careful validation, leakage prevention, and assessment of calibration and clinical utility [50]. Furthermore, an RF model for STEMI patients after PCI demonstrated a high AUC and F1-score in the development dataset, suggesting good discrimination in that specific cohort, but its clinical utility depends on calibration, external validation, and integration into real-world workflows [16]. XGBoost and bagging models have shown favorable discrimination in some datasets, but their apparent stability may be influenced by feature-selection procedures, missing-data handling, hyperparameter tuning, and whether external validation was performed. For example, in a study on first-time AMI patients, a Bagging model achieved an AUC of 0.932 on the test set and 0.893 on external validation. Moreover, the SHAP values clarified the contributions of key variables such as D-dimer, brain natriuretic peptide (BNP), and CS [51,52]. An XGBoost model developed for AMI patients with CS yielded an AUC of 0.835. Although this was slightly lower than that of a logistic Regression model (0.841), the XGBoost model demonstrated greater robustness in handling missing values and imbalanced data [41,52,53]. Neural network models may be useful for processing high-dimensional biomarker data, but their advantages over simpler models are not universal and must be weighed against reduced transparency, higher data requirements, and overfitting risk. For instance, a neural network model based on complete blood count with differential (CBC w/diff) achieved an AUC of 0.788, outperforming traditional clinical scores such as the Sequential Organ Failure Assessment (SOFA) score. After variable selection, its AUC improved to 0.812. Additionally, a Gradient Boosting Classifier model for patients with sepsis and AMI achieved an AUC of 0.82, which was significantly higher than that of traditional scoring systems, confirming the capability of neural networks to integrate data related to multi-organ dysfunction [54,55]. Table 2 (Ref. [9,16,32,42,48,56,57]) summarizes the features of some commonly used models.

**Table 2.** **Representative traditional risk scores and machine-learning models for predicting in-hospital mortality in AMI**.

Category	Model	Setting/population	Key predictors (examples)	Performance	Strengths	Limitations/notes
Traditional score	GRACE	ACS/AMI; multinational registry-derived	Age, HR, SBP, creatinine, Killip class, etc. (8 predictors)	C-statistic ~0.81 (in-hospital death) [9]; AUC = 0.754 in diabetic AMI [48]	Simple; interpretable; widely validated	Lower accuracy in some subgroups (e.g., diabetes) and some Asian cohorts; recalibration may help
Traditional score	TIMI	Mainly STEMI bedside risk stratification	Age, HR, SBP, Killip class, diabetes history, etc.	AUC ~0.75 (in-hospital death) [56]	Very easy; minimal data	Limited discrimination; may underestimate risk in older or multi-organ dysfunction patients
Traditional score	SAPS III	ICU/CCU severity score applied to AMI	Demographics, comorbidities, physiologic variables	AUC = 0.901 in CCU AMI (MIMIC-IV); >LODS/OASIS/SAPS II [57]	Captures global severity; good discrimination/calibration in CCU	Not AMI-specific; higher data burden; less practical outside ICU/CCU
Machine learning	Random Forest	High-dimensional non-linear clinical data (e.g., post-PCI; diabetes)	Clinical + labs (age, HR, SBP, creatinine, enzymes, etc.); SHAP: urine output/anion gap/BUN in diabetic AMI	AUC = 0.976 post-PCI AMI (SCIENCE DB), >XGBoost/AdaBoost/logistic [16]	Strong performance; models interactions; SHAP improves interpretability	Needs external validation; sensitive to data quality/leakage; interpretability still limited
Machine learning	CatBoost	Gradient-boosted trees; robust to categorical/missing data	Age, HR, SBP, creatinine, glucose, HbA1c, etc.	AUC = 0.92 in diabetic STEMI (in-hospital death) [32]	Handles heterogeneous EHR data; high accuracy	Feature-importance/SHAP recommended; domain shift may reduce performance
Deep learning	DNN (Echo)/DL+NLP	Imaging or multi-modal data; can add unstructured EHR text	Echo: LVEF, LVEDD, E/A, etc. (LVEF often key); text features can be added	AUC = 0.912 (echo DNN) [42]	Captures complex patterns; supports multi-modal fusion	High data/compute demand; lower transparency; cross-center generalizability can be challenging

ACS, acute coronary syndrome; AMI, acute myocardial infarction; AUC, area under the curve; BUN, blood urea nitrogen; CCU, coronary care unit; DNN, deep neural network; EHR, electronic health record; GRACE, Global Registry of Acute Coronary Events; HR, heart rate; ICU, intensive care unit; LVEF, left ventricular ejection fraction; LVEDD, left ventricular end-diastolic diameter; NLP, natural language processing; PCI, percutaneous coronary intervention; RF, random forest; SAPS III, Simplified Acute Physiology Score III; SBP, systolic blood pressure; SHAP, Shapley Additive Explanations; STEMI, ST-segment elevation myocardial infarction; TIMI, Thrombolysis in Myocardial Infarction.

Overall, machine-learning models may improve discriminative performance in some settings, particularly when high-dimensional data, nonlinear relationships, and complex subgroup heterogeneity are present. However, these improvements are often modest and highly dependent on data quality, feature availability, validation strategy, and the comparator model. Therefore, higher AUC values should be interpreted cautiously and should not be equated directly with superior clinical utility. In studies focusing on patients with AMI complicated by cardiogenic shock, some machine-learning models reported higher AUC values than traditional logistic regression models; however, the clinical relevance of these differences depends on calibration, decision-curve benefit, reproducibility, and performance in external cohorts [52]. In a study employing a stacking model for patients with NSTEMI, the machine-learning model achieved a higher AUC than logistic regression. However, such findings should be interpreted in the context of study design, sample size, feature selection, risk of overfitting, and the availability of external validation [50]. However, traditional regression-based models and established risk scores retain important advantages in transparency, interpretability, ease of bedside use, and often more straightforward calibration assessment. The GRACE score has demonstrated more stable calibration across multiple studies (with H–L test *p*-values closer to 0.05) [46,47]. Furthermore, the coefficients of logistic regression models can directly quantify the contribution of variables to mortality risk, making them easier for clinicians to understand and accept [4,58]. Additionally, the risk of overfitting in machine-learning models must be mitigated through rigorous cross-validation and external validation. For instance, a LASSO regression model developed for AMI patients complicated by cardiac arrest ensured calibration stability via 1000 bootstrap validations, while a RF model reduced overfitting bias through out-of-bag (OOB) sample validation [59,60] (Fig. 3).

**Fig. 3.**
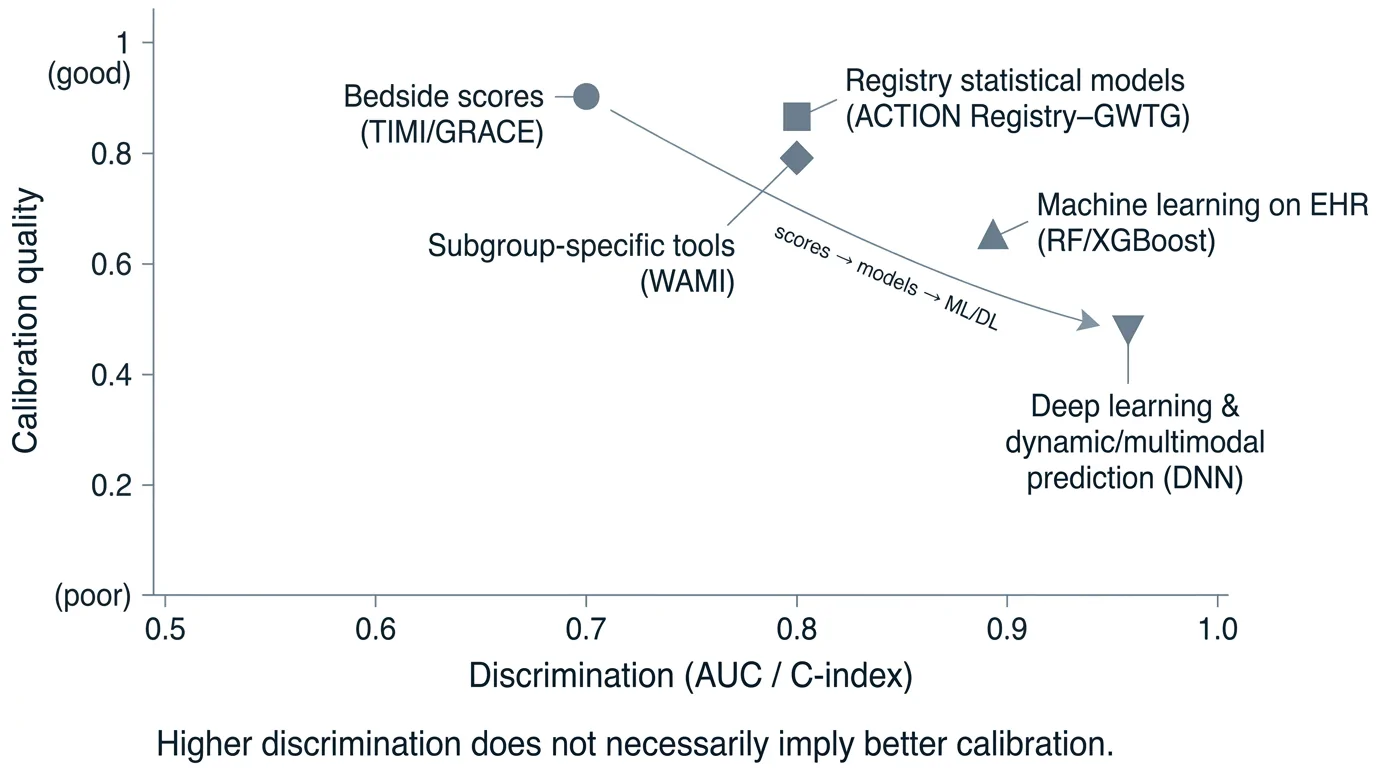
**Conceptual relationship between discrimination and calibration across model families for predicting in-hospital mortality in AMI**.

### 3.2 Methodological Limitations Across Existing Prediction Studies

Despite the increasing number of AMI mortality prediction models, the current evidence base should be interpreted with caution because several methodological limitations are common across studies. First, many models are developed using relatively small, single-center, or highly selected retrospective cohorts, which may inflate apparent performance and reduce generalizability. Second, external validation remains insufficient, and many studies rely primarily on random data splitting or internal cross-validation, which cannot fully evaluate transportability across hospitals, ethnic groups, treatment strategies, or data-collection systems. Third, information leakage may occur when variables measured after clinical decision-making or close to the outcome event are included as predictors, resulting in overly optimistic AUC estimates. Fourth, feature selection is sometimes performed before data splitting or without adequate clinical justification, increasing the risk of overfitting and unstable predictor selection. Finally, missing-data handling, class imbalance, calibration assessment, and decision-curve analysis are inconsistently reported. Therefore, very high AUC values should not be interpreted as definitive evidence of model superiority unless supported by transparent model development, appropriate validation, robust calibration, and demonstrated clinical usefulness.

### 3.3 Clinical Efficacy and Applicability Analysis

The clinical validity of a model is highly dependent on its application context, encompassing patient population (STEMI/NSTEMI, type of comorbidities), treatment strategy (PCI/medical therapy, veno-arterial extracorporeal membrane oxygenation [VA-ECMO] support), and healthcare resources. Models for STEMI patients often require a greater focus on variables related to reperfusion therapy. In addition to procedural characteristics, readily available admission variables may provide important prognostic information. In a large CAMI registry study of 10,482 patients with STEMI undergoing PCI, both lower and higher admission blood pressure levels were associated with increased in-hospital mortality, highlighting the importance of context-specific clinical predictors in early risk stratification [61]. For instance, a prediction model for STEMI patients complicated by CS incorporated treatment-related variables such as post-PCI ejection fraction (EF), the no-reflow phenomenon, and the use of intra-aortic balloon pump (IABP). Its decision curve analysis (DCA) demonstrated significant net benefit within a risk threshold range of 20%–80% [62]. Conversely, a STEMI model based on a multicenter PCI registry in China included variables such as symptom-to-balloon time (SBT) and SYNTAX score. Its clinical decision curve confirmed the model’s value in guiding reperfusion strategy selection [63]. For NSTEMI patients, models that integrate comorbidities and biomarkers are more essential. For example, a nomogram model for non-diabetic NSTEMI patients incorporated easily accessible variables including age, blood urea nitrogen, and albumin. Its DCA curve outperformed traditional risk scores across a risk threshold range of 10%–90% [64]. Furthermore, the improvement in net reclassification index (NRI) of the CAMI-NSTEMI model over the GRACE model (0.3311) was directly translated into adjustments in clinical treatment strategies, such as early PCI for higher-risk patients [65]. Dedicated models are required for subgroup patients with complex comorbidities to ensure clinical validity. For instance, a risk score for AMI patients with ESRD incorporated renal disease-related biomarkers such as CRP and D-dimer. Its AUC (0.895) was significantly higher than that of the GRACE score (0.754), and its DCA curve showed clinical net benefit within a risk threshold range of 30%–70% [66,67]. For AMI patients complicated by CA who received VA-ECMO support, a prediction model incorporated organ function indicators such as lactate and creatinine. Its DCA curve demonstrated significantly greater net benefit than traditional scores at risk thresholds above 20% [68].

Another dimension of clinical effectiveness is the cost-effectiveness of model implementation, encompassing the accessibility of variable acquisition, computational complexity, and time costs. Traditional models, with fewer variables and simpler calculations, are more suitable for rapid risk stratification in emergency settings. The WAMI score requires only eight easily obtainable variables, allowing clinicians to complete the calculation within five minutes using a paper form or a mobile application [31,69]. In contrast, the SMR model based on the VA database utilizes variables automatically extracted from the EHR, eliminating the need for additional testing and making it suitable for real-time risk monitoring in large populations [70]. Although machine-learning models involve more variables, efficient clinical application can still be achieved through EHR integration and automated computation. For instance, the Bagging model for patients with a first AMI incorporates variables such as D-dimer and BNP, which are routine laboratory tests that can be automatically imported for calculation via the Laboratory Information System (LIS) [51]. The nomogram model based on the MIMIC-IV database has been developed into an online calculator, enabling clinicians to obtain real-time risk predictions by inputting just ten variables [71]. Furthermore, some machine-learning models have reduced the number of variables through feature selection techniques, such as LASSO regression. For example, a model for AMI patients with CS incorporates only seven variables, achieving a balance between performance and efficiency [4,41].

A key prerequisite for clinical validity is the external validation and generalizability of a model across populations. Multiple studies have shown that model performance varies significantly across different geographical regions, ethnicities, and healthcare systems, necessitating external validation to ensure its applicability. For instance, the GRACE score demonstrated an AUC of 0.8957 in the Thai PCI registry study and an AUC of 0.926 in a Chinese ACS cohort, indicating good cross-regional stability [46,47]. Similarly, a model for AMI patients complicated by CS was externally validated across two centers (AUCs of 0.826 and 0.783, respectively), confirming its generalizability across different healthcare institutions [68,72]. Furthermore, model updating and recalibration are crucial for maintaining its long-term clinical validity. For example, the VA’s SMR model maintains performance comparable to the original model in the 2017–2019 cohort through periodic coefficient updates [73]. In the Thai PCI registry study, updating the coefficients of the GRACE score improved its AUC from 0.8957 to 0.9118, further optimizing its clinical validity [46,74].

As summarized in Table 3, traditional models such as GRACE, TIMI, ACTION Registry-GWTG, and CAMI remain clinically valuable because of their simplicity, interpretability, and relatively broad validation. Machine-learning models may achieve higher discrimination in selected datasets, especially when high-dimensional EHR or multimodal data are available. However, their apparent performance advantage should be interpreted cautiously because calibration, external validation, decision-curve benefit, information leakage, and workflow feasibility are inconsistently reported. Therefore, model selection should depend not only on AUC, but also on calibration, clinical usefulness, interpretability, local transportability, and implementation feasibility.

**Table 3.** **Summary comparison of major models for predicting in-hospital mortality in AMI**.

Model	Model type	Target population/data source	Validation	Discrimination	Calibration/clinical utility	Main strengths	Main limitations
GRACE	Traditional risk score	ACS/AMI; multinational registry-derived	Widely externally validated	C-statistic generally around 0.80–0.90; lower in some high-risk subgroups	Calibration varies across populations; local recalibration may be required	Simple, interpretable, and widely used	May underperform in specific subgroups and contemporary cohorts
TIMI	Traditional bedside score	STEMI/NSTEMI risk stratification	Broadly validated	AUC generally lower than GRACE in many studies	Calibration reporting is limited in some cohorts	Very simple and suitable for rapid bedside use	Limited variables; may underestimate risk in elderly or complex patients
ACTION Registry-GWTG	Registry-based statistical model	Large-scale AMI registry population	Large multicenter validation	C-statistic approximately 0.87–0.88	Good overall performance; calibration should be checked in local populations	Strong registry basis and clinically interpretable predictors	Requires local recalibration when transferred across healthcare systems
CAMI	Registry-based statistical model	Chinese AMI registry population	Internal and multicenter validation in China	C-statistic approximately 0.84	Designed for the Chinese AMI population; calibration may be better locally	Region-specific applicability; useful for Chinese clinical settings	Generalizability outside China requires further validation
WAMI	Subgroup-specific score	Female AMI patients	External validation reported	C-statistic approximately 0.87	Calibration and utility depend on the validation cohort	Tailored to female patients	Limited to a specific population; broader validation is needed
Random forest	Machine-learning model	AMI cohorts with structured EHR or registry variables	Often internal validation; some external validation	AUC may be high in selected datasets	Calibration and DCA are inconsistently reported	Captures nonlinear relationships and interactions	Risk of overfitting, information leakage, and limited interpretability
XGBoost/LightGBM/CatBoost	Machine-learning model	High-dimensional structured clinical data	Mostly retrospective validation	AUC commonly favorable but dataset-dependent	Requires calibration assessment and external validation	Handles complex interactions and missing data better than some methods	Sensitive to feature selection, hyperparameter tuning, and domain shift
Deep learning models	Deep learning model	Large EHR, imaging, or multimodal datasets	Limited external or prospective validation	AUC may be favorable in selected datasets	Clinical utility remains uncertain without prospective evaluation	Potential for multimodal prediction	High data demand, poor transparency, and implementation barriers

ACS, acute coronary syndrome; AMI, acute myocardial infarction; AUC, area under the receiver operating characteristic curve; CAMI, China Acute Myocardial Infarction Registry; DCA, decision curve analysis; EHR, electronic health record; GRACE, Global Registry of Acute Coronary Events; STEMI, ST-segment elevation myocardial infarction; NSTEMI, non-ST-segment elevation myocardial infarction; TIMI, Thrombolysis in Myocardial Infarction; WAMI, Women Acute Myocardial Infarction in-Hospital Mortality score.

## 4. Recalibration Strategies for In-Hospital Mortality Prediction Models

### 4.1 The Necessity and Core Principles of Model Recalibration

The clinical application of in-hospital mortality prediction models for AMI relies on their accuracy in the target population. However, discrepancies between model development and real-world application scenarios often lead to prediction bias, making recalibration a crucial step in model optimization. The necessity for model recalibration primarily stems from the heterogeneity of population characteristics. AMI patients from different regions, ethnicities, and age groups exhibit significant variations in baseline risk, treatment strategies, and comorbidity profiles. For instance, the CAMI-NSTEMI score, developed from the CAMI registry, demonstrated superior calibration compared to the GRACE score among Chinese NSTEMI patients [75]. However, its performance may deviate in other populations (e.g., Southeast Asian or European/American cohorts) due to differences in patient baseline characteristics, such as the distribution of comorbidities and PCI utilization rates. Secondly, the evolution of treatment paradigms continuously impacts model performance. With the widespread adoption of PCI techniques, the application of novel antiplatelet agents (e.g., ticagrelor), and the improvement of cardiac rehabilitation systems, the prognosis of AMI patients has significantly improved. Consequently, early models (e.g., the TIMI score) may overestimate the mortality risk for contemporary patients [76]. Furthermore, data quality and the handling of missing values can also lead to model miscalibration. For example, laboratory indicators (e.g., troponin, creatinine) for AMI patients in the MIMIC-IV database exhibit varying degrees of missingness. Inconsistent imputation methods between model development and practical application scenarios may affect prediction accuracy [77].

The core principles of model recalibration should center on clinical utility and statistical rigor. First, target population adaptability requires that the recalibration process be based on real-world data from the target population, not solely on the original development set. For example, the deep learning model (DAMI) developed from the Korean Myocardial Infarction (KorMI) registry demonstrated excellent discrimination in external validation but still required calibration based on the specific patient characteristics of different hospitals [78]. Secondly, the principle of dynamism emphasizes the need for regular model updates. Due to changes in AMI treatment strategies (e.g., the use of SGLT2 inhibitors) and shifts in patient risk profiles, models should be periodically monitored and recalibrated according to changes in the local population and clinical setting using the most recent data [79]. Third, the principle of transparency mandates that the recalibration process clearly report the adjusted parameters, methods, and validation results to enable clinicians to understand the reliability of model predictions. For instance, model updating should transparently report the adjusted parameters, updating methods, and validation results so that clinicians can judge the reliability of recalibrated predictions [80,81]. Finally, the principle of minimal intervention suggests prioritizing simple parameter adjustments (e.g., intercept recalibration) over model reconstruction to optimize performance while preserving model interpretability [80].

Calibration should be understood as a multidimensional property rather than a single statistical test. Calibration-in-the-large evaluates whether the average predicted risk is systematically higher or lower than the observed event rate in the target population. A positive or negative overall mismatch indicates that the model may require intercept recalibration. In contrast, the calibration slope reflects whether predicted risks are too extreme or too moderate across the risk spectrum. A slope below 1 usually suggests overfitting or overly extreme predictions, whereas a slope above 1 suggests insufficient separation of predicted risks. Thus, recalibration should improve both overall risk agreement and risk separation across clinically relevant risk groups. In addition, calibration should be evaluated using complementary approaches, including calibration plots, calibration-in-the-large, calibration slope, and the Brier score. The Hosmer–Lemeshow test may be reported as a supplementary measure, but it should not be used as a standalone assessment because it is sensitive to sample size, grouping strategy, and event frequency [82].

### 4.2 Implementation Path and Effect Evaluation of Recalibration

The implementation challenges discussed in this review are relevant across healthcare systems. These challenges include external validation, local recalibration, EHR integration, model governance, prospective evaluation, and performance monitoring. Their practical solutions may differ by region because AMI care pathways, access to PCI-capable hospitals, data standardization, reimbursement policies, and digital health infrastructure vary substantially.

The implementation of recalibration should follow a standardized process to ensure the reliability and reproducibility of the results [81]. First, during the data preparation phase, complete data from the target population must be collected, including all variables used in the original model development and outcome events such as in-hospital mortality [83]. For the recalibration of the GRACE score, core variables such as patient age, heart rate, systolic blood pressure, and creatinine levels need to be obtained. Special attention should be paid to the handling of missing values during data cleaning: When missingness is non-negligible, multiple imputation is preferred over complete-case analysis to reduce bias; sensitivity analyses are recommended when missingness is substantial [84,85,86]. Second, the model evaluation phase requires simultaneous analysis of the model’s discrimination and calibration. During model evaluation, discrimination and calibration should be assessed together, using metrics such as the C-statistic, calibration plot, calibration-in-the-large, calibration slope, and Brier score [83]. If the model exhibits good discrimination but poor calibration, parameter adjustment methods should be selected; if discrimination is also poor, model updating is necessary. Third, during the calibration implementation phase, an appropriate method should be chosen based on the evaluation results. If the model only exhibits an overall risk bias, such as predicted mortality being higher than the observed value, intercept recalibration is applied; if the bias increases with rising risk levels, slope recalibration or piecewise recalibration is used. For machine-learning models, Platt Scaling or Isotonic Regression can be employed for calibration: Platt Scaling adjusts predicted probabilities via logistic regression and is suitable for smaller sample sizes; Isotonic Regression fits a monotonically increasing calibration curve using a non-parametric method and is suitable for large sample data [39]. Fourth, the validation phase requires assessing the calibration effect through internal and external validation. In the multi-center validation of the CAMI score within China, the Bootstrap method was used for 1000 repeated samplings to ensure the stability of the recalibrated model across different subsets [35].

The evaluation of recalibration effectiveness should be conducted from two dimensions: statistical performance and clinical utility. Statistical performance assessment includes three aspects: discrimination, calibration, and reclassification improvement. Discrimination is still measured using the C-statistic or AUC. For example, the AUC of the GRACE score increased from 0.75 to 0.81 after recalibration. Calibration is evaluated through the Hosmer-Lemeshow test, calibration curve, and Brier score. For instance, the Brier score of the ACTION Registry-GWTG model in the validation set was 0.046, indicating a small prediction error [87]. Reclassification improvement is measured using the net reclassification index (NRI) and the integrated discrimination improvement index (IDI). For example, the CAMI-NSTEMI score showed improved risk classification compared with the GRACE score in Chinese NSTEMI patients [75]. Clinical utility assessment should consider the risk stratification capability and clinical decision impact after model recalibration. Risk stratification capability is measured by the difference in actual mortality rates among different risk groups. A well-calibrated model should be able to stratify patients into low-risk (mortality <1%), intermediate-risk (1%–5%), and high-risk (>5%) groups, with significant differences in mortality rates between these groups. The impact on clinical decisions is assessed using DCA to evaluate the net benefit of the model across different threshold probabilities. Unlike AUC or calibration statistics, DCA directly addresses whether model-guided decisions would improve clinical outcomes compared with strategies of treating all patients or treating none. For AMI in-hospital mortality prediction, this is particularly relevant because risk estimates may influence intensive care unit (ICU) admission, invasive monitoring, early mechanical circulatory support, or intensified antithrombotic and reperfusion strategies. For example, the HAMIOT score demonstrated a significantly higher net benefit than the GRACE score at threshold probabilities between 5% and 20% [34]. Furthermore, the operational feasibility of the recalibrated model should be evaluated, including the ease of variable acquisition and computational complexity. Prediction models based on routine blood test indicators, such as white blood cell count (WBC) and hemoglobin (Hb), are more suitable for application in primary care hospitals due to the easy accessibility of these variables [88]. Therefore, the clinical usefulness of a recalibrated model should not be inferred from statistical performance alone. A clinically useful model should provide well-calibrated absolute risk estimates, separate patients into actionable risk categories, improve decision-making within clinically plausible threshold ranges, and rely on variables that can be obtained rapidly and reliably in emergency or coronary care settings. In this context, DCA, workflow feasibility, interpretability, and prospective impact on clinical decisions are necessary complements to AUC, calibration statistics, and reclassification indices.

### 4.3 Challenges and Future Directions of Recalibration

Several challenges limit the routine use of recalibrated models. First, data accessibility issues: certain model variables (e.g., peak troponin) are difficult to obtain in primary care hospitals, preventing the comprehensive implementation of recalibration [89]. Second, model heterogeneity: recalibration methods differ across various models (e.g., GRACE vs. CAMI), lacking a unified standard. Third, insufficient dynamism: most models still employ static recalibration and fail to achieve real-time updates. Future directions include: (1) developing automated recalibration tools: utilizing EHR systems to collect data in real-time and automatically trigger model evaluation and calibration [90]; (2) integrating multi-source data: combining radiomics (e.g., echocardiographic parameters) and genomics data to improve the personalized predictive capability of models [91]; (3) exploring adaptive recalibration approaches that allow models to be updated using recent local data and clinical feedback; and (4) conducting cost-effectiveness analyses: evaluating the impact of recalibrated models on healthcare resource allocation (e.g., ICU bed utilization) [92].

In summary, the recalibration of in-hospital mortality prediction models for AMI serves as a critical bridge connecting model development with clinical application. By selecting appropriate recalibration methods, adhering to standardized implementation pathways, and integrating clinical needs, the accuracy and utility of these models can be significantly improved, providing robust support for risk stratification and individualized treatment of AMI patients. Future efforts should further explore automated and adaptive recalibration strategies to address the evolving landscape of clinical practice and patient characteristics.

### 4.4 Practical Barriers and Governance Requirements for Clinical Implementation

The clinical implementation of AMI mortality prediction models requires more than favorable retrospective performance. First, models must be integrated into electronic health record systems through automated data extraction, standardized variable definitions, real-time calculation, and workflow-compatible visualization. Models relying on manual input or variables unavailable at the time of decision-making are unlikely to be clinically useful. Second, regulatory approval and model governance are needed, including model registration, version control, privacy protection, accountability, and clear rules for clinician override. Third, prospective trials or pragmatic implementation studies are required to determine whether model-guided care improves outcomes, resource allocation, decision efficiency, or cost-effectiveness. Finally, deployed models require continuous monitoring because performance drift may occur as patient populations, treatment strategies, coding practices, and data quality change over time. Periodic assessment of discrimination, calibration, subgroup performance, and clinical impact should therefore be accompanied by local recalibration or updating when necessary.

## 5. Conclusion

Prediction models for in-hospital mortality in AMI have evolved from traditional statistical methods to machine-learning and artificial-intelligence approaches. However, improvements in discrimination are often incremental, and model value should not be judged by AUC alone. Future work should prioritize calibration, interpretability, external validation, EHR-based integration, prospective implementation studies, governance frameworks, performance monitoring, and timely recalibration. These efforts are essential for safe, sustainable, and clinically useful adoption of AMI mortality prediction models across diverse healthcare systems.
